# Functional Characterization of Human T Cell Hyporesponsiveness Induced by CTLA4-Ig

**DOI:** 10.1371/journal.pone.0122198

**Published:** 2015-04-10

**Authors:** Yrina Rochman, Masashi Yukawa, Andrey V. Kartashov, Artem Barski

**Affiliations:** 1 Division of Allergy and Immunology, Cincinnati Children’s Hospital Medical Center and Department of Pediatrics, College of Medicine, University of Cincinnati, Cincinnati, Ohio, United States of America; 2 Division of Immunobiology, Cincinnati Children’s Hospital Medical Center and Department of Pediatrics, College of Medicine, University of Cincinnati, Cincinnati, Ohio, United States of America; 3 Division of Human Genetics, Cincinnati Children’s Hospital Medical Center and Department of Pediatrics, College of Medicine, University of Cincinnati, Cincinnati, Ohio, United States of America; University of Melbourne, AUSTRALIA

## Abstract

During activation, T cells integrate multiple signals from APCs and cytokine milieu. The blockade of these signals can have clinical benefits as exemplified by CTLA4-Ig, which blocks interaction of B7 co-stimulatory molecules on APCs with CD28 on T cells. Variants of CTLA4-Ig, abatacept and belatacept are FDA approved as immunosuppressive agents in arthritis and transplantation, yet murine studies suggested that CTLA4-Ig could be beneficial in a number of other diseases. However, detailed analysis of human CD4 cell hyporesponsivness induced by CTLA4-Ig has not been performed. Herein, we established a model to study the effect of CTLA4-Ig on the activation of human naïve T cells in a human mixed lymphocytes system. Comparison of human CD4 cells activated in the presence or absence of CTLA4-Ig showed that co-stimulation blockade during TCR activation does not affect NFAT signaling but results in decreased activation of NF-κB and AP-1 transcription factors followed by a profound decrease in proliferation and cytokine production. The resulting T cells become hyporesponsive to secondary activation and, although capable of receiving TCR signals, fail to proliferate or produce cytokines, demonstrating properties of anergic cells. However, unlike some models of T cell anergy, these cells did not possess increased levels of the TCR signaling inhibitor CBLB. Rather, the CTLA4-Ig–induced hyporesponsiveness was associated with an elevated level of p27^kip1^ cyclin-dependent kinase inhibitor.

## Introduction

During activation, T cells integrate multiple signal inputs from APCs and the cytokine milieu. Of the different co-stimulatory receptors that are expressed on the surface of naïve cells, CD28 is the primary molecule that is required for full T cell activation[1,2]. CD28 interacts with B7 ligands on the surface of APCs and signals via PDK1/PKC-θ, PI3K/AKT, and RAS/ERK-1/2 cascades, leading to increased activation of AP-1 and NF-κB transcriptional factors[2]. This co-stimulatory signaling can be blocked by CTLA4-Ig, a fusion protein composed of the extracellular domain of CTLA-4 and Fc domain of IgG1. CTLA-4, an inhibitory receptor on T cells, can interact with high affinity with B7 molecules on APCs[2–4]. The ability of CTLA-4 to bind B7 receptors with high affinity was exploited to develop a CTLA4-Ig protein that prevents CD28-B7 interaction by blocking B7 receptors. In mice, the co-stimulatory blockade during priming promotes generation of dysfunctional T cells via induction of T cell anergy[1,5]. The ability of CTLA4-Ig to induce immunosuppression has been illustrated in murine models of transplantation, arthritis, and diabetes[5–9]. In murine models of asthma, administration of CTLA4-Ig either prior to sensitization or before challenge was shown to reduce lung inflammation and eosinophilia[10–12].

In clinic, abatacept and belatacept, two pharmacologically modified forms of CTLA4-Ig, are FDA approved for treatment of rheumatoid arthritis and in kidney transplantation, respectively[3,4,8,9,13]. These biologicals have been used in more than 140 completed and ongoing clinical trials in autoimmune diseases (arthritis, uveitis, alopecia areata, type I diabetes, SLE), transplantation, GVHD, and asthma. Despite being generally well tolerated, CTLA4-Ig had a mixed record of success: efficacy was shown in arthritis, and the use in SLE and type 1 diabetes was also promising, but in some of the other immunological diseases, such as asthma, the use of abatacept was less beneficial[14–18]. This result in humans contrasted with the murine asthma studies, in which CTLA4-Ig strongly reduced lung inflammation[11,12,19]. This mixed efficacy record underscores the need for better mechanistic understanding of CTLA4-Ig action, whereas the discrepancies between human and mouse results stress the need to study these mechanisms specifically in the human system.

Given the clinical importance of CTLA4-Ig, it is surprising that the mechanisms responsible for its action, particularly in humans, have not been fully understood. Accordingly, we performed functional and transcriptional analysis of CTLA4-Ig’s effect on the activation of human naïve T cells in an *ex vivo* mixed lymphocyte culture model [5,20,21]. Consistent with the current understanding of signaling networks, the blockade of CD28 co-stimulation during TCR priming decreased activation of AKT, cJUN, and NF-κB but did not alter other pathways, such as phosphorylation of zeta-chain–associated protein kinase 70 (ZAP70) and MAPKs and nuclear translocation of NFATs. Cells activated in the presence of CTLA4-Ig became anergic and were not able to proliferate or produce cytokines during secondary activation. Notably, we did not detect increased expression of E3 ubiquitin ligases, diacylglycerol kinase alpha (DGKA), or early growth response (EGR) family proteins in anergic cells compared to fully activated cells during primary or secondary response of T cells. This suggested that TCR signaling was not inhibited in the anergized cells. Indeed, anergic cells expressed the same level of CD3 and CD28 as effector cells, and their hyporesponsiveness could be overcome by IL-2. However, human anergic cells had an elevated level of p27^kip1^ cyclin-dependent kinase inhibitor, which was likely responsible for the decreased cellular proliferation of anergic cells[22–24].

## Materials and Methods

### Generation of human anergic, effector, and regulatory T cells

Blood samples were obtained from Hoxworth Blood bank. Samples were de-identified, and the study was conducted under an exemption provided by the Cincinnati Children’s Hospital Medical Center (CCHMC) IRB. PBMCs were depleted of CD45RO^+^, CD8^+^, and CD25^+^ cells using biotinylated antibodies (BioLegend) and IMag streptavidin beads (BD Biosciences) and stimulated in 6-well plates at 8x10^6^ cells/2 ml with soluble αCD3 (2 μg/ml, OKT-3, BioXCell) and αCD28 (1 μg/ml, BD Biosciences) or CTLA4-Ig (7.5 μg/ml, BioXCell) in RPMI 1640 supplemented with 10% FBS, penicillin/streptomycin, L-glutamine, and β-mercaptoethanol. To generate regulatory T cells (Treg cells), αCD3, αCD28, IL-2 (100 U/ml, Roche), and TGF-β (10 ng/ml, Pepro Tech.) were added. Cells were harvested after 4 days and washed in PBS, and CD4^+^ T cells were purified by negative selection using the human CD4 EasySep kit (STEMCELL Technologies). Purified CD4^+^ cells were rested for 4–5 days in medium and then re-stimulated with soluble αCD3 (2 μg/ml) and αCD28 (1 μg/ml) or other stimuli for the indicated time periods.

To check primary response, depleted cells were primed, and CD4^+^ T cells were purified by positive selection using human CD4 Dynabeads (Invitrogen) at the indicated time points prior to RNA isolation or lysate preparation.

### Flow cytometry

The surface staining was done in live cells using the following anti-human antibodies: FITC-αCD3, FITC-αCD45RA (Milteney Biotech), FITC-αCD8 (HIT8a), PE-αCD25 (BC96), APC-αCD127 (A019D5) (BioLegend), PE-Cy5 or APC-αCD4 (RPA-T4), APC-Cy7-αCD45RA (OX-33), PE-αCD28 (CD28.2), PE-Cy7-αCD45RO (UCHL1), APC-αCD62L (DREG-56), and FITC-αCD69 (FN50) (BD Biosciences). Intracellular FITC-FOXP3 (206D) staining was performed according to the FOXP3 kit instructions (BioLegend).

To detect protein phosphorylation, cells were fixed with Cytofix Buffer (BD Biosciences) for 10 minutes in 37°C and permeabilized in 90% methanol overnight in -20°C. The next day, cells were re-suspended in staining buffer and labeled with PE-αpZAP70 or PE-αpERK1/ERK2 antibodies (BD Biosciences) for 1 hour at room temperature.

For apoptosis measurement, cells were resuspended in 100 μl of annexin V buffer and stained with FITC-annexin V + 7-AAD (BD Biosciences) for 15 minutes at room temperature.

Proliferation was examined by labeling cells with 2.5 μM CFSE (Life Technologies) for 8 minutes at room temperature and monitoring CFSE dilution in CD4^+^-gated cells at the indicated days after treatment.

### Suppression assay

Rested CD4^+^ cells (2.5x10^4^; naïve, EML, anergic, or Treg cells) were co-cultured with allogeneic naïve CFSE-labeled responder CD4^+^ T cells at ratio 1:1 in 96-well round bottom plates in the presence of Dynabeads Human T-activator αCD3/αCD28 (Invitrogen) (0.1 μl of beads per 100 μl of medium) for 4 days. The percentage of divided CFSE-labeled responder cells (D) was calculated to each sample, and the suppressive capacity of CD4 subsets (EML, anergic, or Treg cells) toward proliferation of CFSE-labeled responders in co-culture (D_subset_) was assessed relative to the baseline proliferation of CFSE-labeled responder cells in the presence of naïve subset (D_naive_) by the equation: [% Suppression = 100 x (D_naïve_-D_subset_)/ D_naïve_].

### Cytokine detection

For intracellular cytokine detection, cells were treated with 500 ng/ml phorbol 12,13-dibutyrate (PDBU) (Sigma) and 1 μM ionomycin (Calbiochem) in the presence of monensin (BioLegend) for 2 hours. Cells then were fixed and permeabilized using the Cytofix/Cytoperm kit (BD Biosciences) and stained with PE- αTNF-α (MAB11), FITC-αIFN-γ (4S.B3), and APC-αIL-2 (MQ1-17H12) monoclonal antibodies (BioLegend).

To measure cytokine secretion, cells were activated as indicated for primary response, and supernatants were collected after 15 hours. For re-stimulation experiments, cells were left resting or were activated with plate-bound αCD3 (10 μg/ml) and soluble αCD28 (2 μg/ml) in the presence of blocking αCD25 (2.5 μg/ml) and αCD122 (1 μg/ml) (both from R&D Systems) for 24 hours. ELISA was performed using capture and biotinylated detector antibodies against human IFN-γ (BioLegend) and IL-2 (R&D Systems).

### Western blot

Cytoplasmic and nuclear fractions of purified CD4^+^ cells were extracted using Nuclear Extract Kit (Active Motif). For preparation of total lysates, RIPA buffer was used. Lysates were resolved on Novex 4–20% Tris-Glycine gels (Invitrogen) and immunoblotted with antibodies to phospho-AKT, AKT, phospho-p42-44, phospho-p38, p38, NFAT1, NFAT2, cJun, NF-κB, CBLB, p27^kip1^, PARP1 (all from Cell Signaling), and β-actin (Sigma).

### RNA isolation, quantitative real-time PCR, and RNA-Seq

RNA was extracted with the Aurum Total RNA Mini Kit (Bio-Rad) and reverse-transcribed with the iScript cDNA Synthesis kit (Bio-Rad). All primers and probes were purchased from IDT, and quantitative real-time PCR was performed on the 7900HT Fast Real-Time PCR System (ABI).

A modified dUTP method was used for preparation of RNA-Seq libraries[25]. Briefly, mRNA was purified from total RNA with the Dynabeads mRNA Purification Kit (Invitrogen) and reverse-transcribed with SuperScript III (Invitrogen). The second strand was created by DNA Polymerase 1 (NEB) in the presence of RNase H and a dNTP mix containing dUTP instead of dTTP. The cDNA was fragmented on a Covaris sonicator, and end repair and A-tailing were performed using the Quick Blunting kit and exo^-^ Klenow fragment of DNA polymerase I, respectively (NEB). Illumina genomic adapters were ligated, and the sample was size-fractionated (~170–400 bp) on E-Gel EX 2% agarose (Invitrogen). After uracil-DNA glycosylase (UDG) treatment (NEB) and a final PCR amplification step with Illumina primers (18 cycles), the cDNA libraries were size-fractionated (~175–400 bp) on a 2% agarose gel, quantified using a Qubit 2.0 fluorometer and dsDNA HS kit (Invitrogen), and sent for sequencing on a Illumina HiSeq 2000 sequencing system resulting in ~10 million reads per sample. RNA-Seq data are available from GEO database (accession # GSE64712)

RNA-Seq data analysis was performed using the BioWardrobe Experiment Management System (www.biowardrobe.com, [26]). Briefly, reads were mapped to the hg18 genome and RefSeq-based transcriptome using RNA-STAR (v. 2.4.0c [27]) and assigned to the RefSeq genes using a BioWardrobe algorithm. Differentially expressed genes with DESEQ (v. 1, [28]) p value <0.05 and RPKM>1 in at least one time point were analyzed. Results were interpreted in the context of biological processes and molecular functions, as well as pathways, through the use of ToppGene Suite[29] for gene list functional enrichment.

## Results

### Effects of co-stimulatory blockade on activation of naïve T cells

**Fig 1:**
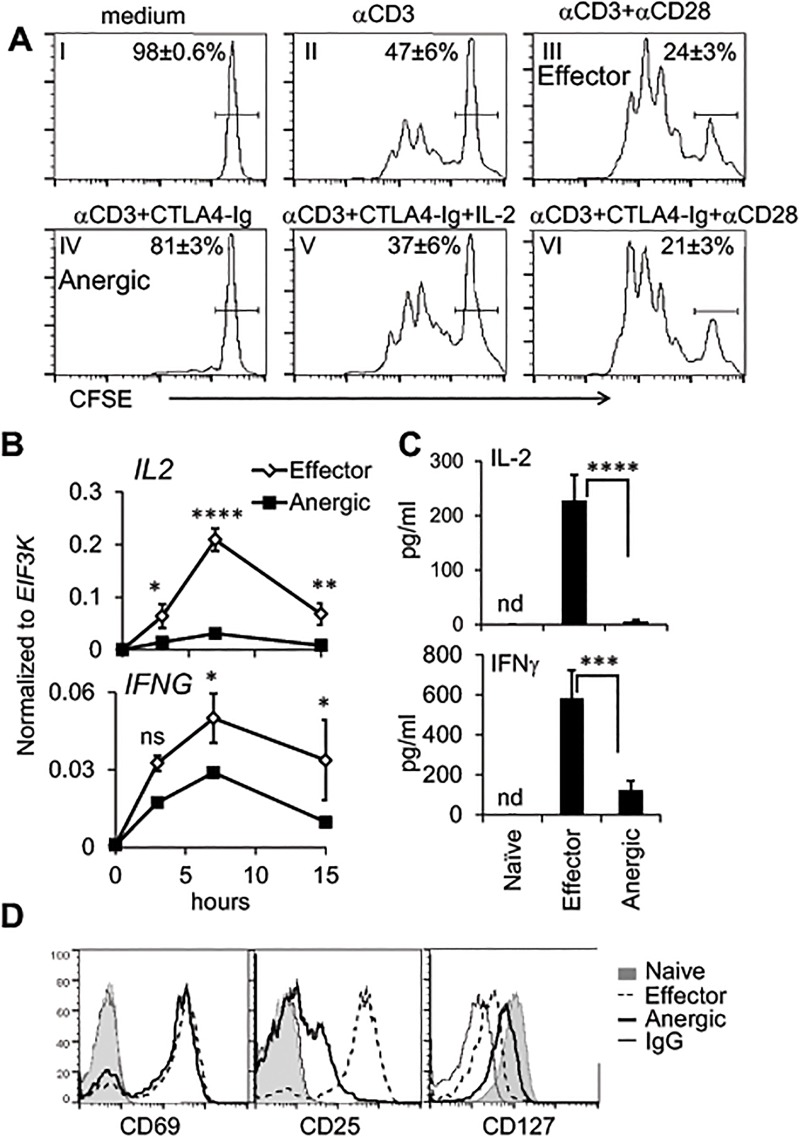
Phenotypic characteristics of activated CD4^+^ T cells during co-stimulatory inhibition. **A.** Human naïve CD4^+^ cells were labeled with CFSE and cultured in medium (plot I) or activated with soluble αCD3 (2 μg/ml) (plots II-VI) in the presence or absence of αCD28 (1 μg/ml), CTLA4-Ig (7.5 μg/ml), and/or IL-2 (100 U/ml) as indicated for 4 days. CFSE intensity in CD4-gated cells was analyzed by FACS. Shown is the mean of the percentage of non-dividing cells ± SEM (n = 6). **B-D.** Human naïve CD4^+^ cells were left resting or were activated for the indicated times by soluble αCD3 antibodies (2 μg/ml) and autologous APCs in the presence of soluble αCD28 (1 μg/ml) (Effector) or CTLA-4-Ig fusion protein (7.5 μg/ml) (Anergic). **B.** Kinetics of *IL2* and *IFNG* mRNA expression, normalized to *EIF3K*, (n = 5) and **C.** levels of cytokine secretion (15 hours, n = 11) were evaluated by qRT-PCR or ELISA, respectively; nd, not detected**. B-C.** The data are presented as mean ± SEM. *p<0.05, **p<0.01, ***p<0.001, ****p<0.0001; ns, not significant. **D.** The presence of surface receptors on CD4^+^ cells were analyzed 1 day (CD69) or 4 days (CD25 and CD127) after cell activation. Shown is one representative from 5 independent experiments.

Co-stimulatory signals enhance and prolong contact between T cells and APCs and synergize with TCR signaling to control normal activation of T cells[2,30]. Therefore, in order to mimic a natural co-stimulatory environment, we performed activation of naïve T cells in an *ex vivo* system in the presence of autologous APCs, as described previously[5,20]. Human PBMCs were depleted of CD8^+^, CD45RO^+^, and CD25^+^ cells, leaving only naïve CD4^+^ T cells and most of the APCs in culture (S1A and S1B Fig). The purity of naïve cells within the CD4^+^ population was more than 95% on the basis of CD62L and CD45RA markers (S1C Fig). Cells were then activated with soluble αCD3 antibody in the absence (Fig 1A, plot II) or presence of αCD28 (plot III, “Effector”) or CTLA4-Ig fusion protein (plot IV, “Anergic”) for 4 days. Co-stimulatory blockade led to a dramatic decrease in proliferation (Fig 1A, plot II and III vs. IV). Addition of αCD28 restored co-stimulation and reversed the inhibitory effect of CTLA4-Ig, suggesting that lack of proliferation is due to an intrinsic T cell defect caused by the lack of co-stimulation rather than an effect of CTLA4-Ig on APCs (Fig 1A, plot IV vs. VI vs. III). The reduction in T cell proliferation in the presence of CTLA4-Ig was also completely reversed by the addition of IL-2 (Fig 1A plot IV vs. V vs. II). Inhibition of co-stimulation by CTLA4-Ig resulted in a marked reduction of IL-2 and IFN-γ mRNA and protein levels (Fig 1B and 1C, respectively), as well as IL-2RA (CD25) expression (Fig 1D). Although activation resulted in a decreased expression of IL-7RA (CD127) in both effector and anergic conditions (Fig 1D), the decrease was less pronounced in anergic conditions. However, CD69 elevation was not affected by CTLA4-Ig, demonstrating efficient TCR signaling (Fig 1D).

**Fig 2:**
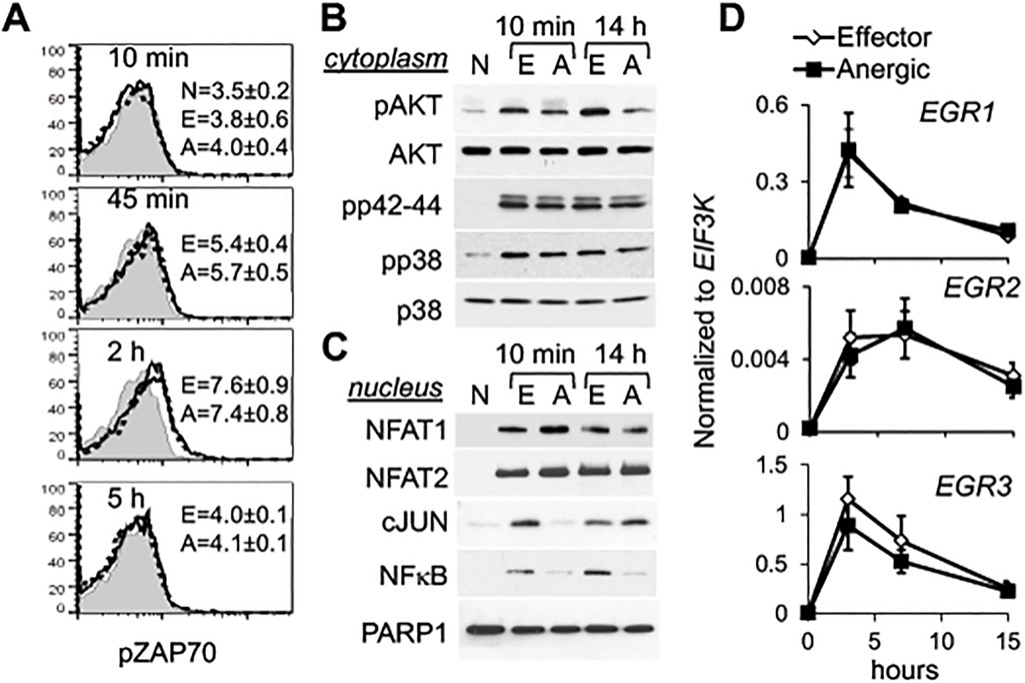
Molecular characteristics of CD4^+^ T cells activated in the presence of co-stimulatory inhibition. Naïve CD4^+^ T cells were activated as in Fig 1B. **A.** Phosphorylation of ZAP70 in CD4^+^ T cells stimulated for the indicated time periods was analyzed by FACS. Shown is one representative experiment with the mean of fluorescence intensity for each population ± SEM from 5 independent experiments. Gray plot is naïve cells (N, unstimulated); dotted line is effector cells (E, αCD3 + αCD28); solid line is anergic cells (A, αCD3 + CTLA4-Ig). **B.** Activation of AKT and MAPKs in cytoplasm and **C.** translocation of transcription factors to the nucleus during activation were examined by western blot (cytoplasmic AKT and p38 and nuclear PARP1 were used as loading controls). Each experiment was repeated at least 3 times. **D.** mRNA expression of indicated genes was measured by real-time PCR (n = 5). Data are presented as mean ± SEM.

Indeed, the abrogation of proliferation and of cytokine production in αCD3/CTLA4-Ig–treated cells was not due to a defect in TCR signaling itself as demonstrated by unaltered ZAP70 phosphorylation (Fig 2A), activation of MAPK pathways (phosphorylated ERK1/2 and p38) (Fig 2B), and NFAT nuclear translocation (Fig 2C). Similarly, induction of the NFAT target genes *EGR1/2/3*[31] was not changed by co-stimulatory blockade (Fig 2D). The expression of negative regulators of TCR signaling, such as *DGKA* and *CBLB*, was also unaffected (data not shown). In contrast, AKT phosphorylation was diminished in the absence of CD28 co-stimulation, and this decrease was sustained at least 14 hours after activation (Fig 2B). Notably, the absence of co-stimulation strongly decreased but did not abolish activation of cJUN and NF-κB (Fig 2C), confirming that both of these pathways are co-induced by TCR and CD28 signals[2].

### Kinetics of transcriptional profile induced by CTLA4-Ig during CD4 T cell activation

**Fig 3:**
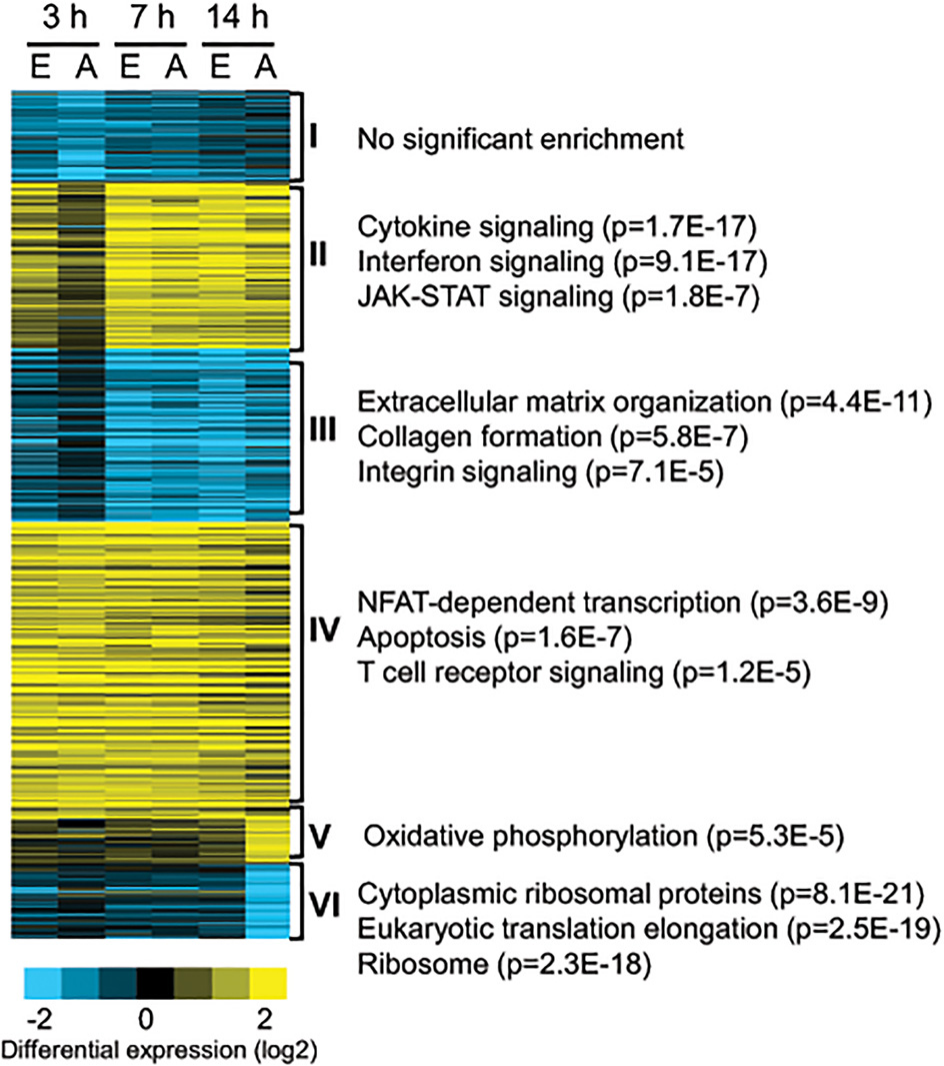
Kinetics of gene expression induced in the presence or absence of CTLA4-Ig in TCR-activated CD4^+^ T cells. Naïve CD4^+^ T cells were activated with αCD3+APCs in the presence or absence of CTLA4-Ig for 0, 3, 7, and 14 hours. CD4 cells were purified and subjected to RNA preparation and RNA-Seq analysis. Data shown are K-mean clustering of genes differentially expressed during activation compared to 0 hours with p value <0.05 and RPKM>1 in at least one condition. Selected gene ontology pathways overrepresented in each cluster are shown to the right together with the corresponding p-value.

To better understand the mechanism of CTLA4-Ig action during T cell activation, we analyzed the kinetics of gene expression in human CD4 cells stimulated by αCD3 and APCs in the presence or absence of CTLA4-Ig (Fig 3). In both conditions, CD4 cells upregulated a large group of genes targeted by TCR signals (cluster IV), confirming intact activity of TCR downstream pathways. Interestingly, major differences between cell populations were observed during the first few hours of activation (cluster II and III, 3 hours). Thus, at early time points, only naïve cells activated by αCD3 in the presence of co-stimulation were able to upregulate genes encoding cytokines and cytokines receptors, such as *IL2*, *CSF2*, *LTA*, *IL2RA*, *IL15RA*, and *IL12RB2*, and transcription factors (TFs) that are important in immune response—*BATF*, *IRFs*, *MYB*, *BHLHE40*, *REL*, and *TBX21* (cluster II). CD4 cells activated in the presence of CTLA4-Ig had a delayed response with lower levels of induction of these genes at later time points (Fig 3, cluster II effector [E] vs. anergic [A]). Similarly, genes downregulated following T cell activation were reduced slower in the presence of CTLA4-Ig treatment (cluster III, 3 hours). A large group of these genes was involved in regulation of cell motility and migration (S1 Table, Biological Process: GO:0048870 and GO:0016477). Surprisingly, CD4 cells stimulated in the presence of CTLA4-Ig showed uniquely expressed or reduced genes at later time points (cluster V and VI, 14 hours). Interestingly, CTLA4-Ig treatment significantly downregulated the expression of genes that are responsible for the activity of translational machinery. This alteration may restrict cell activation processes, inhibit cell proliferation, and promote anergic cell generation. More comprehensive analysis of the functional activity of these genes will be needed to elucidate a mechanism of CTLA4-Ig action on anergic cell generation.

### Human CD4^+^ cells activated in the presence of CTLA4-Ig become hyporesponsive

**Fig 4:**
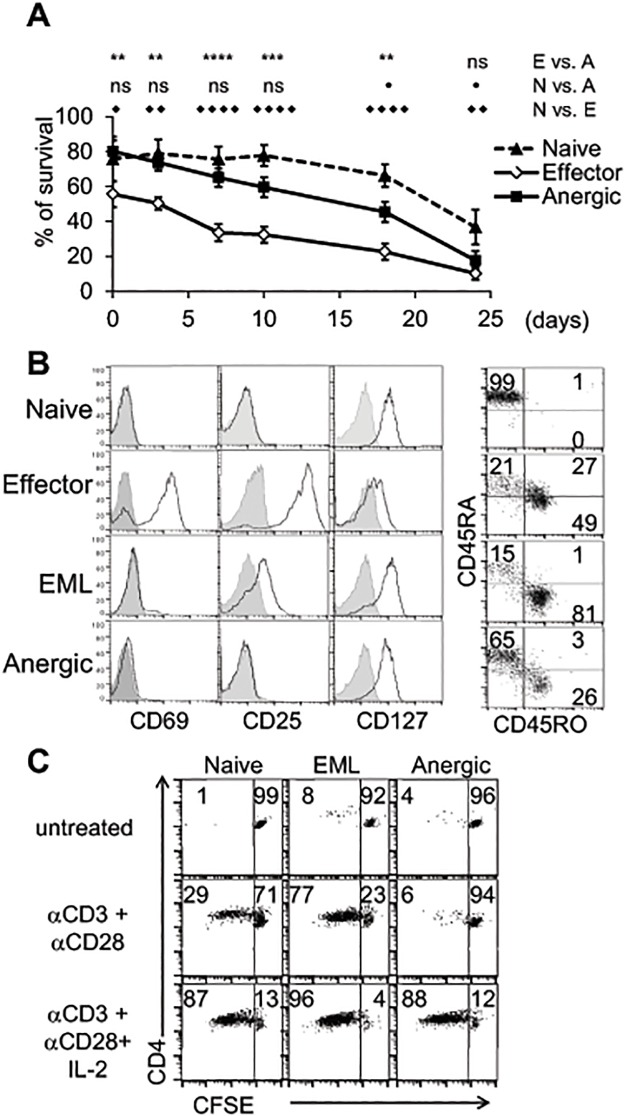
Survival, surface marker expression, and proliferation rate of anergic, effector/memory-like (EML), and naïve CD4^+^ T cells. Naïve, effector, and anergic cells were purified after 4 days of primary activation in the presence of autologous APCs. **A.** Cells were cultured in medium with 10% FBS and stained with Annexin V and 7-AAD to define apoptotic vs. live cells (n = 10). The data are presented as mean ± SEM. (**p<0.01, ***p<0.001, ****p<0.0001; ns, not significant). **B.** Fluorescence intensity of the indicated cell surface markers was measured on naïve, 4 days activated (effector), or 4 days activated and 3 days rested (EML and anergic) cells. The histograms show the expression of cell surface markers (solid line) and IgG control (gray plot). **C.** Purified populations of CD4^+^ T cells were rested for 4 days in medium, labeled with CFSE, and left untreated or re-stimulated with plate-bound αCD3 (5 μg/ml) + soluble αCD28 (2 μg/ml) ± IL-2 (100 U/ml). Numbers represent the percentage of dividing or non-dividing cells. **B-C.** Shown is one representative experiment from at least 3 experiments.

In order to understand long-term effects of CTLA4-Ig on human T cells, we studied T cell survival and response to secondary activation in the cells that were activated in the presence or absence of co-stimulatory blockade. Effector cells had an increased apoptotic rate immediately after activation (0 days) and decreased survival during the following three weeks (Fig 4A). Anergic cells demonstrated lower levels of cell death than effectors on day 0, however, this population still had a higher apoptotic rate than naïve cells (Fig 4A).

**Fig 5:**
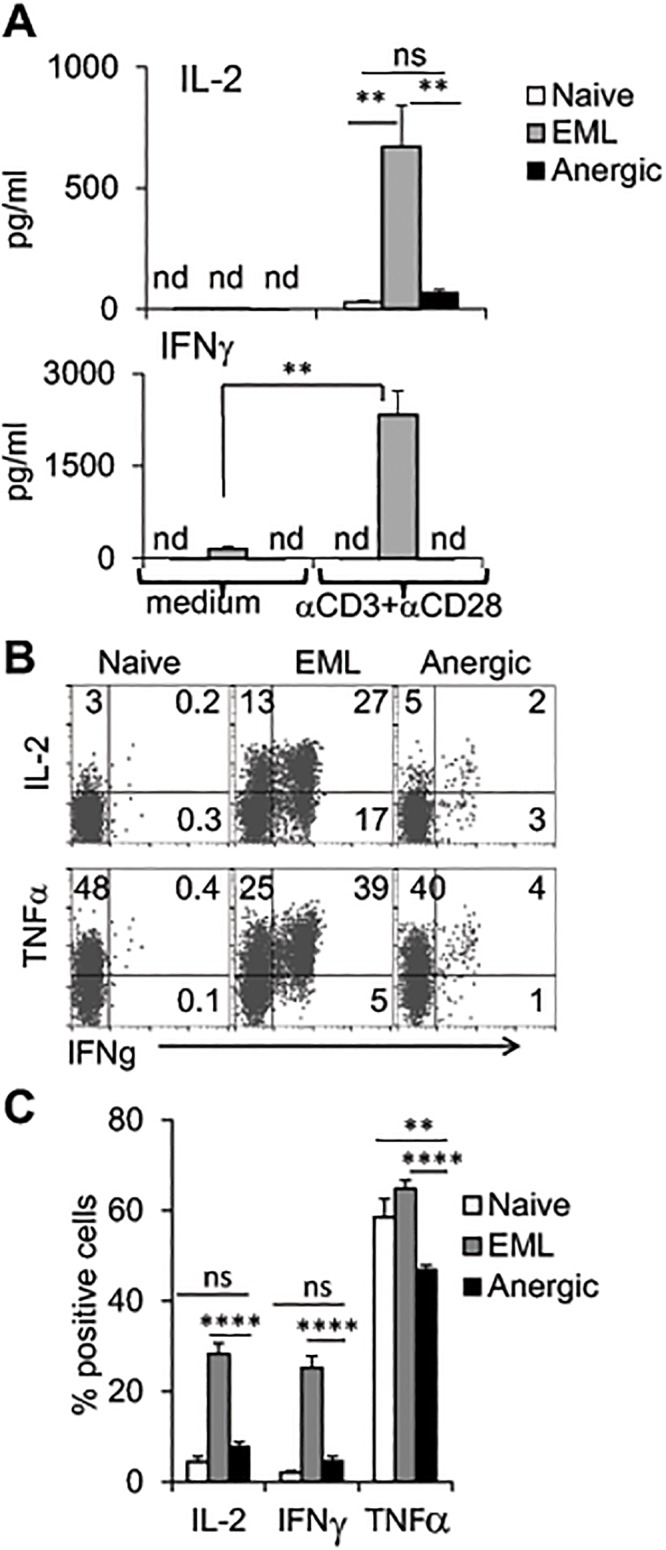
Anergic cells have a defect in cytokine production. **A-B.** Naïve CD4^+^ cells were activated as in Fig 1, purified, and rested in the medium for 4–5 days. **A.** Purified and rested (4 days) naïve, EML, and anergic cells were activated with plate-bound αCD3 and soluble αCD28 for 24 hours in the presence of αCD25 + αCD122 to prevent IL-2 usage. ELISA for the indicated cytokines was performed, and data are presented as mean ± SD of 3 independent experiments (**p<0.01; nd, not detected; ns, not significant). **B-C.** Cells were treated with PDBU and ionomycin in the presence of monensin for 2 hours. The intracellular staining of cytokines was evaluated by FACS. **C.** Average percentage of cytokine-positive cells from a group of 16 naïve, 39 EML, and 39 anergic cell samples (**p<0.01, ****p<0.0001). The data are presented as mean ± SEM.

Since effector cells began expressing CD45RO instead of CD45RA and lost activation properties (see below) after 3 days of rest (Fig 4B and 4C), thus demonstrating similarity to memory cells, we will henceforth refer to these rested effector cells as effector/memory-like (EML) cells. After 3 days of rest in the medium, both EML and anergic cells had reduced expression of the activation markers CD69 and CD25, but upregulated CD127 (Fig 4B, EML and anergic vs. effector), were not dividing (Fig 4C, upper row), and were not producing cytokines (Fig 5A, medium). Notably, most anergic cells continued to express CD45RA, demonstrating similarity to the surface phenotype of naïve CD4^+^ cells (Fig 4B). However, in contrast to naïve or EML cells, anergic cells were unable to proliferate under αCD3+αCD28 activation (Fig 4C, middle row). As had been reported previously[32], addition of IL-2 to the culture rescued proliferation of anergic cells, as well as increased proliferation of EML and naïve cells (Fig 4C). These results showed that although anergic cells have an intrinsic defect that reduces their responsiveness, this defect might be overcome by additional of IL-2.

Next we measured the ability of CD4 cells primed in the presence of CTLA4-Ig to produce cytokines upon secondary activation. As expected, these cells had decreased ability to produce cytokines following their re-stimulation with plate-bound αCD3 + αCD28 (Fig 5A, anergic vs. EML, 24 hours) or by short stimulation with PDBU + ionomycin (Fig 5B and 5C, 2 hours). The percentages of IL-2–, IFN-γ–, and TNF-α–producing cells were significantly lower in this cell population compared to EML cells (Fig 5B and 5C). Interestingly, both IL-2 and IFN-γ were produced mostly by memory-acquired phenotype EML cells (CD45RA^-^CD45RO^+^), whereas CD45RA^-^CD45RO^+^ anergic cells did not produce IFN-γ and showed lower expression of IL-2 compared to EML cells (S2 Fig). In summary, human naïve CD4^+^ cells activated in the presence of APCs, αCD3, and CTLA4-Ig fusion protein acquire characteristics of anergic cells: high viability but low proliferation rate and cytokine production in response to secondary activation.

### Anergic cells do not have the phenotypic or functional characteristics of regulatory T cells

**Fig 6:**
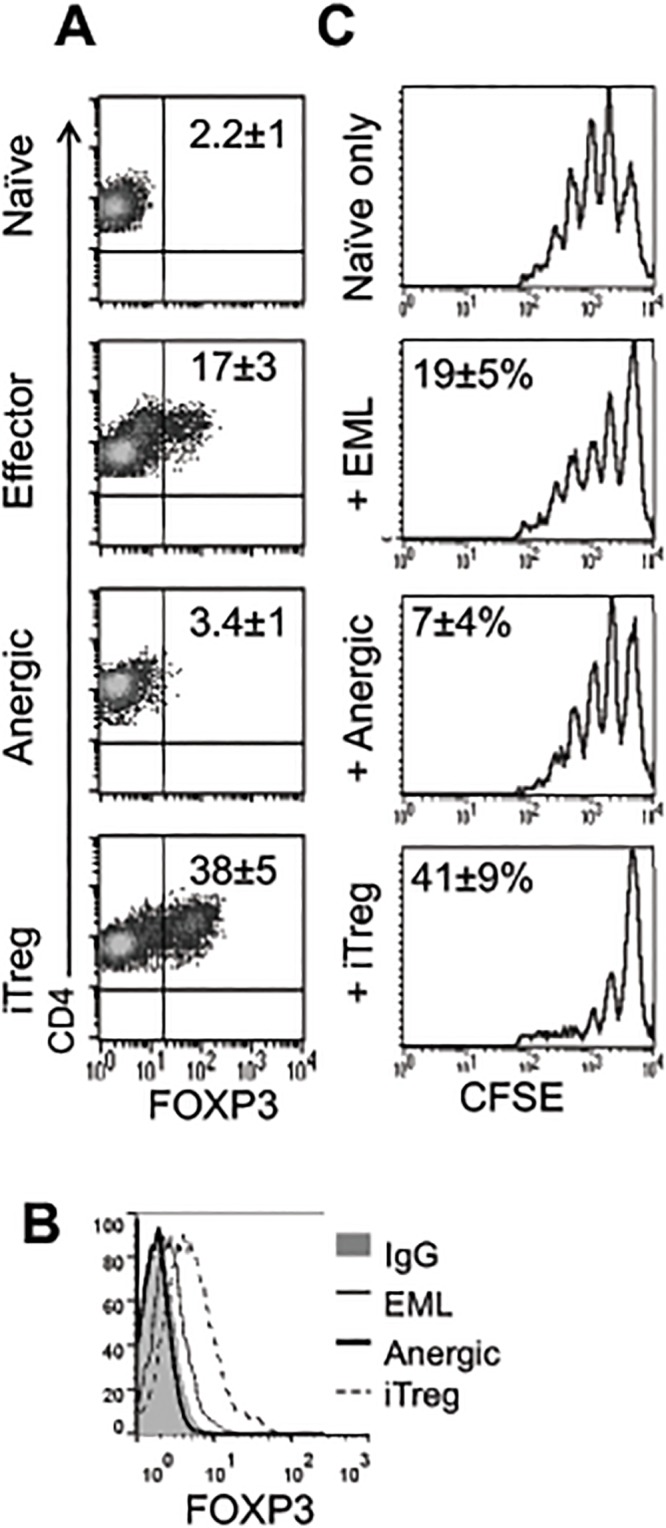
CD4^+^ T cells activated in the presence of CTLA4-Ig do not have properties of Treg cells. **A-C**. Naïve cells were activated to generate effector or anergic cells as in Fig 1. For generation of regulatory T cells (Treg cells), αCD3, αCD28, TGF-β, and IL-2 were added for the first activation. **A.** The expression of FOXP3 in CD4^+^ T cells was measured on day 4 of activation or **B.** after an additional 3 days of rest in the medium. Numbers are percent of CD4^+^FOXP3^+^ cells ± SEM (n = 11) **C.** Each group of CD4^+^ T cells was purified 4 days after activation, rested for 3 days in medium, and co-activated with freshly purified CFSE-labeled allogeneic naïve CD4^+^ cells in the presence of αCD3 + αCD28–coated beads (0.1 μl of beads per 100 μl of medium) for 4 days. The proliferation rate of CFSE-labeled naïve cells was analyzed by FACS. Shown is the average ± SEM percent inhibition of naïve cell proliferation (n = 6). EML, effector/memory-like T cells; iTreg, induced regulatory T cells.

Given that T cells generated by activation in the presence of costimulatory blockade exhibit some of the properties of Treg cells, namely poor proliferation and absence of IL-2 secretion, we decided to compare these two cell populations. For this purpose, FOXP3, IL-10, and CD25 expression, as well as suppressive activity of anergic cells, was compared with those of induced Treg (iTreg) cells. Anergic cells did not express FOXP3 during the first TCR activation (Fig 6A) or after 3 days of rest (Fig 6B) and showed only a very slight upregulation of *IL10* mRNA (S3 Fig). Similarly, they expressed only a low level of IL-2RA (CD25) after TCR activation (Fig 1D) and lost it after 3 days of rest (Fig 4B). Further, anergic cells demonstrated a minimal inhibitory effect on naïve CD4^+^ T cell proliferation compared to the inhibitory effect of iTreg cells or even EML cells (Fig 6C). Together these results demonstrate that the anergic cells generated in our system did not acquire phenotypic or functional properties of Treg cells.

### Human anergic cells generated in the presence of CTLA4-Ig express normal levels of E3 ubiquitin ligases but an elevated level of p27^kip1^

**Fig 7:**
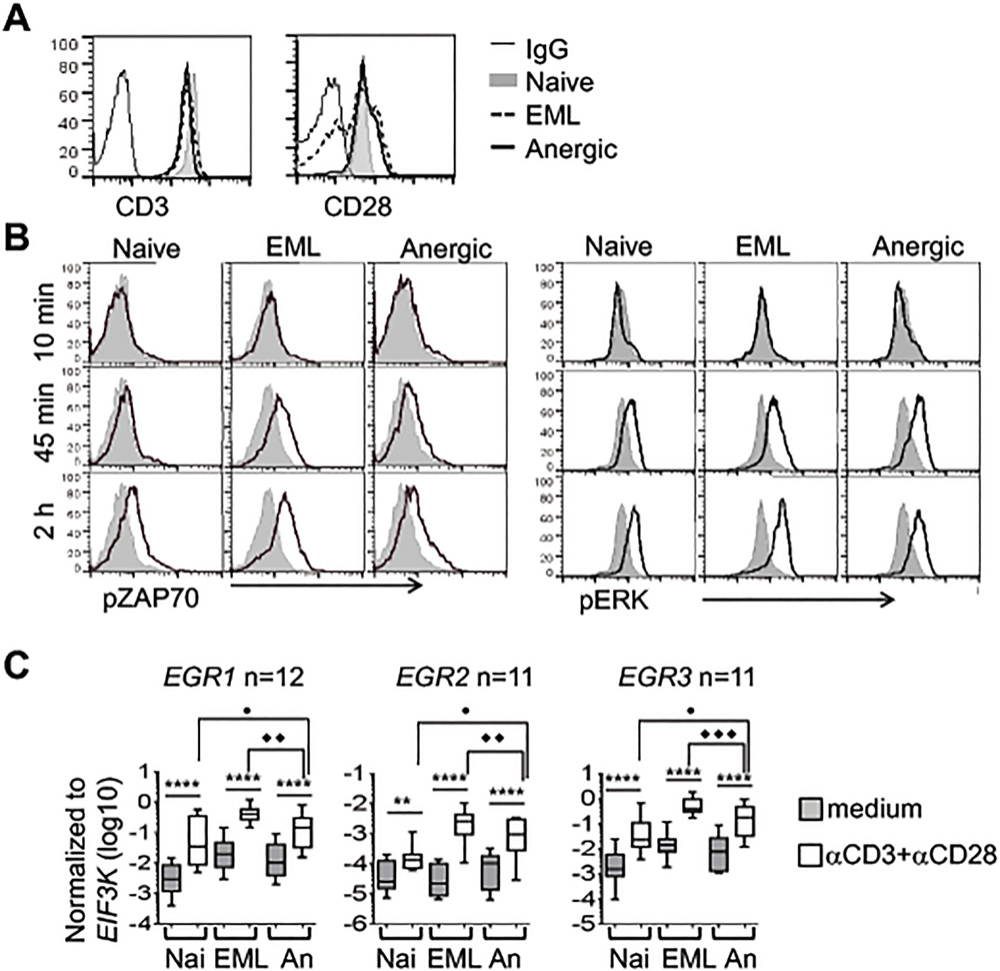
Anergic cells can receive TCR signaling. Naïve cells were activated to generate effector or anergic cells as in Fig 1 and rested for 4–5 days. **A.** The surface expression of CD3e and CD28 was analyzed by FACS. **B.** The phosphorylation of ZAP70 (left panel) and ERK1/2 (right panel) was measured in unstimulated (gray plot) and αCD3 + αCD28–stimulated (solid line) naïve, effector/memory-like (EML), and anergic cells at the indicated time points. Shown is one representative experiment out of 4 independent experiments. **C.** Rested cells were not activated or were activated with soluble αCD3 + αCD28 for 4 hours. mRNA expression of the indicated genes was measured by qRT-PCR (Box and whiskers plot). The data are presented as mean ± SEM (*p<0.05, **p<0.01, ***p<0.001, ****p<0.0001). An, anergic cells; EML, effector/memory-like T cells; Nai, naïve cells.

What could be behind the failure of anergic cells to proliferate and produce cytokines? The defect of proliferation of anergic cells was not due to decreased expression of CD3 and CD28 receptors or alterations in TCR signaling. Anergic cells expressed normal levels of both receptors compared with naïve and EML cells (Fig 7A). They were able to activate effectors downstream of TCR, such as ZAP70 and ERK (Fig 7B) and to elevate mRNA levels of EGR transcription factors (Fig 7C), although to a slightly lower level than EML cells.

**Fig 8:**
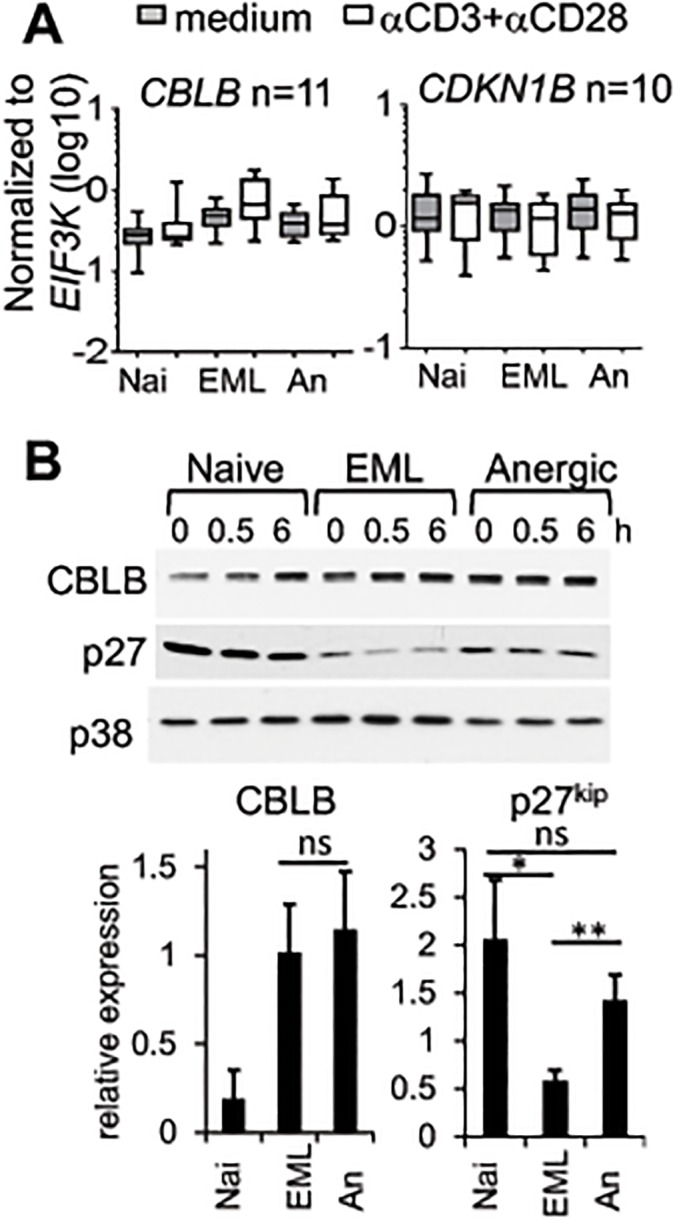
Human anergic cells have increased level of p27^kip1^. **A-B.** Naïve, EML, and anergic cells were left untreated or stimulated with soluble αCD3 + αCD28, and **A.** mRNA level after 4 hours (Box and whiskers plot) or **B.** protein level of CBLB and p27^kip1^ after 0, 0.5, and 6 hours were measured. The lower panel represents the relative protein expression of CBLB and p27^kip1^ to p38 as loading control from 6 (CBLB) and 10 (p27^kip1^) independent experiments at 0 hours (*p<0.05, **p<0.01; ns, not significant). The data are presented as mean ± SEM. An, anergic cells; EML, effector/memory-like T cells; Nai, naïve cells.

We next measured the expression of an inhibitor of TCR signaling, CBLB, and a negative regulator of proliferation, p27^kip1^ (cyclin-dependent kinase inhibitor 1B [CDKN1B]), proteins reported to be involved in the maintenance of anergic phenotype in other systems[22–24,33]. Both anergic and EML cells had similar mRNA levels of *CBLB* (Fig 8A). Similar results were obtained on the protein level of CBLB (Fig 8B). The expression of other E3 ligases, *ITCH* and *GRAIL*, also was not different between naïve, EML, and anergic cells (data not shown). *CDKN1B* (p27^kip1^) mRNA was expressed at a similar level in all three cell types (Fig 8A), but naïve and anergic cells had a significantly higher p27^kip1^ protein level than EML cells (Fig 8B). The difference between mRNA and protein results is not surprising since post-transcriptional regulation of p27^kip1^ expression has been demonstrated previously[34]. Consistent with previous findings in human anergic T-cell clones[26], our results showed that the addition of IL-2 during anergic T cell activation reduces p27^kip1^ protein level (S4 Fig). These results suggest that T cell hyporesponsiveness is not due to E3 ligases inhibiting TCR signaling but possibly due to a heightened threshold for mitogenic signaling, represented by increased levels of p27^kip1^, as discussed previously for the murine system[22] and human T cell clones[24].

## Discussion

In various anergy models, multiple factors were reported to be responsible for anergy induction. It has been shown that E3 ubiquitin ligases, the DGK family, p27^kip1^, Ikaros, EGR2, and EGR3 proteins contribute to the anergy program in murine models[21–24,33,35–39] and may be called “anergic factors”. In our model of human anergic T cells induced by CTLA4-Ig, we did not observe overexpression of these molecules compared to fully activated cells, with the exception of p27^kip1^. In order to test whether the discrepancy between the models is due to differences between humans and mice, we also induced anergy in human T cells by ionomycin treatment, as was described previously[33]. Ionomycin upregulated the expression of *EGR2*, *EGR3*, and *CBLB* but not *EGR1*, in agreement with results obtained in murine cells[33,40] (S5 Fig). These data suggest that in addition to possible inter-species differences in the anergy induction, different signals may induce the anergic state by diverse mechanisms.

In our system, the anergic phenotype seems to be maintained by increased level of p27^kip1^, whereas addition of IL-2 during anergic cell activation decreases p27^kip1^ protein level. p27^kip1^ is an inhibitor of G1/S CDK/cyclin complexes, including cyclin D/CDK4, A/CDK2, and E/CDK2[41]. Downregulation of p27^kip1^ was previously associated with the ability of cells to proliferate[34]. In mice, abundance of p27^kip1^ protein was strongly correlated with anergy induction, whereas p27^kip1^ knockout prevented induction of T cell anergy in a murine costimulatory blockade model[22–24]. On the other hand, p27^kip1^ was not required for induction of anergy in a clonal anergy model[42]. Altogether, these results support the notion that the hyporesponsive phenotype can be maintained by different mechanisms and suggest that other, possibly downstream, effectors may play a role. Further work will be required to fully understand the pathways affected in anergic cells.

Our results do not provide a complete explanation for the mixed efficacy record of CTLA4-Ig in clinical trials, and further work is clearly needed. One possibility is that although CTLA4-Ig renders T cells hyporesponsive, this anergy can be overcome in the presence of IL-2. This provides a potential explanation for the lack of clinical benefit in some of the CTLA4-Ig clinical trials discussed above[14–18]. Alternatively, CTLA4-Ig may affect aspects of immunity not recapitulated in our system: e.g. T cell help to B cells. A more stable form of peripheral tolerance, induction of iTreg, is likely required to inhibit autoimmune or atopic reactions in the long term. However, our data show that in addition to preventing effector response, co-stimulatory blockade also inhibits FOXP3 expression and diminishes T cell suppressive activity (Fig 6). Similar results were reported in a murine allograft transplantation model[43]. This is not surprising given that anergic cells fail to produce IL-2 and express a low level of IL-2RA, revealing a lack of the IL-2 signaling that is critical for iTreg induction. Notably, the lower expression of IL-2RA and higher expression of IL-7RA that we observed in cells activated in the presence of CTLA4-Ig (Fig 1D) can be also explained by low IL-2 production[44–46].

To summarize, we established and performed molecular and functional characterization of a human model of T cell anergy induced by co-stimulatory blockade. This model utilizes physiological signaling pathways and, in this respect, is highly relevant to the endogenous processes that generate anergic cells. Our results provided a detailed analysis of the effect of CTLA4-Ig on the generation of human anergic T cells. We demonstrated that CTLA4-Ig might promote hyporesponsivness by mechanisms that are distinct from those of murine models of anergy or anergy induced by ionomycin. Given the therapeutic role of CTLA4-Ig, we believe that this model may serve as a tool to more deeply understand the phenomenon of human anergy and the effects of abatacept and belatacept on human T cell functions.

## Supporting Information

S1 Fig Experimental approach (A) and cell isolation (B-C). Human PBMCs were depleted of CD45RO, CD8, and CD25 cells, and the purity of population was detected by the indicated markers **B.** gated on total cell population and **C.** gated on CD4^+^ T cells.
(EPS)

S2 Fig Anergic cells with memory phenotype produce low level IFN-γ and IL-2. EML and anergic cells were rested for 3 days and then were treated with PDBU and ionomycin in the presence of monensin for 2 hours. Surface staining of CD45RA and CD45RO and intracellular staining of cytokines were evaluated by FACS. Cells first were gated on CD45RA^+^CD45RO^-^ and CD45RA^-^CD45RO^+^ populations. Shown is 1 of 2 representative experiments.
(EPS)

S3 Fig Anergic cells express low level of IL-10. Kinetics of *IL10* mRNA expression, normalized to *EIF3K* (n = 3). The data are presented as mean ± SEM (*p<0.05; ns, not significant).
(EPS)

S4 Fig IL-2 decreases protein level of p27^kip1^. Human anergic CD4^+^ cells were rested for 3 days and then left unstimulated or stimulated with plate-bound αCD3 and soluble αCD28 in the presence or absence of IL-2 (100 U/ml) for 24 hours. The protein level of p27^kip1^ and β-actin were measured. The lower panel represents the relative protein expression of p27^kip1^ to β-actin as loading control. The experiment was performed twice, and data are presented as mean **±** SD (***p<0.001).
(EPS)

S5 Fig Comparison with ionomycin model. Pre-activated human CD4^+^ cells were rested for 3 days and then stimulated with 1 **μ**M of ionomycin for the indicated periods of time. mRNA levels of *EGR1*, *EGR2*, *EGR3*, and *CBLB* were normalized to expression of the housekeeping gene *EIF3K* and calculated relative to time 0 (before ionomycin treatment). The experiment was performed twice, and data are presented as mean **±** SD.
(EPS)

S1 Table Functional enrichment analysis. Selected pathways enriched in RNA-Seq–based gene clusters were identified using Toppgene (https://toppgene.cchmc.org).
(XLSX)

## References

[1] Choi S, Schwartz RH (2007) Molecular mechanisms for adaptive tolerance and other T cell anergy models. Semin Immunol 19: 140–152. 17400472 10.1016/j.smim.2007.02.005 PMC2045643

[2] Chen L, Flies DB (2013) Molecular mechanisms of T cell co-stimulation and co-inhibition. Nat Rev Immunol 13: 227–242. 10.1038/nri3405 23470321 PMC3786574

[3] Wekerle T, Kurtz J, Bigenzahn S, Takeuchi Y, Sykes M (2002) Mechanisms of transplant tolerance induction using costimulatory blockade. Curr Opin Immunol 14: 592–600. 12183158 10.1016/s0952-7915(02)00378-3

[4] Wekerle T, Grinyó JM (2012) Belatacept: from rational design to clinical application. Transpl Int 25: 139–150. 10.1111/j.1432-2277.2011.01386.x 22151353

[5] Wells AD, Walsh MC, Bluestone JA, Turka LA (2001) Signaling through CD28 and CTLA-4 controls two distinct forms of T cell anergy. J Clin Invest 108: 895–903. 11560959 10.1172/JCI13220 PMC200935

[6] Webb LM, Walmsley MJ, Feldmann M (1996) Prevention and amelioration of collagen-induced arthritis by blockade of the CD28 co-stimulatory pathway: requirement for both B7-1 and B7-2. Eur J Immunol 26: 2320–2328. 8898940 10.1002/eji.1830261008

[7] Lenschow DJ, Ho SC, Sattar H, Rhee L, Gray G, et al (1995) Differential effects of anti-B7-1 and anti-B7-2 monoclonal antibody treatment on the development of diabetes in the nonobese diabetic mouse. J Exp Med 181: 1145–1155. 7532678 10.1084/jem.181.3.1145 PMC2191918

[8] Linsley PS, Nadler SG (2009) The clinical utility of inhibiting CD28-mediated costimulation. Immunol Rev 229: 307–321. 10.1111/j.1600-065X.2009.00780.x 19426230

[9] Silva M V, Machado JR, Rocha LP, Castellano LR, Reis MA, et al (2012) CD28 Family and Chronic Rejection: “To Belatacept...and Beyond!.” J Transplant 2012: 1–14. 10.1155/2012/203780 PMC3376773 22720132

[10] Keane-Myers A, Gause WC, Linsley PS, Chen SJ, Wills-Karp M (1997) B7-CD28/CTLA-4 costimulatory pathways are required for the development of T helper cell 2-mediated allergic airway responses to inhaled antigens. J Immunol 158: 2042–2049. 9036947

[11] Padrid P, Mathur M, Li X, Herrmann K, Qin Y, et al (1998) CTLA4Ig inhibits airway eosinophilia and hyperresponsiveness by regulating the development of Th1/Th2 subsets in a murine model of asthma. Am J Respir Cell Mol Biol 18: 453–462. 9533932 10.1165/ajrcmb.18.4.3055

[12] Van Oosterhout AJM, Hofstra CL, Shields R, Chan B, Van Ark I, et al (1997) Murine CTLA4-IgG Treatment Inhibits Airway Eosinophilia and Hyperresponsiveness and Attenuates IgE Upregulation in a Murine Model of Allergic Asthma. Am J Respir Cell Mol Biol 17: 386–392. 9308926 10.1165/ajrcmb.17.3.2679

[13] Caporali R, Bugatti S, Cavagna L, Antivalle M, Sarzi-Puttini P (2014) Modulating the co-stimulatory signal for T cell activation in rheumatoid arthritis: could it be the first step of the treatment? Autoimmun Rev 13: 49–53. 10.1016/j.autrev.2013.06.008 23777823

[14] Merrill JT, Burgos-Vargas R, Westhovens R, Chalmers A, D’Cruz D, et al (2010) The efficacy and safety of abatacept in patients with non-life-threatening manifestations of systemic lupus erythematosus: Results of a twelve-month, multicenter, exploratory, phase IIb, randomized, double-blind, placebo-controlled trial. Arthritis Rheum 62: 3077–3087. 10.1002/art.27601 20533545

[15] Orban T, Bundy B, Becker DJ, DiMeglio L, Gitelman SE, et al (2011) Co-stimulation modulation with abatacept in patients with recent-onset type 1 diabetes: a randomised, double-blind, placebo-controlled trial. Lancet 378: 412–419. 10.1016/S0140-6736(11)60886-6 21719096 PMC3462593

[16] Mayer L, Kaser A, Blumberg RS (2012) Dead on arrival: understanding the failure of CTLA4-immunoglobulin therapy in inflammatory bowel disease. Gastroenterology 143: 13–17. 10.1053/j.gastro.2012.05.015 22626501 PMC3392152

[17] Sandborn WJ, Colombel J-F, Sands BE, Rutgeerts P, Targan SR, et al (2012) Abatacept for Crohn’s disease and ulcerative colitis. Gastroenterology 143: 62–69.e4 10.1053/j.gastro.2012.04.010 22504093

[18] Parulekar AD, Boomer JS, Patterson BM, Yin-Declue H, Deppong CM, et al (2013) A randomized controlled trial to evaluate inhibition of T-cell costimulation in allergen-induced airway inflammation. Am J Respir Crit Care Med 187: 494–501. 10.1164/rccm.201207-1205OC 23292882 PMC5448510

[19] Keane-Myers A, Gause WC, Linsley PS, Chen SJ, Wills-Karp M (1997) B7-CD28/CTLA-4 costimulatory pathways are required for the development of T helper cell 2-mediated allergic airway responses to inhaled antigens. J Immunol 158: 2042–2049. 9036947

[20] Tan P, Anasetti C, Hansen JA, Melrose J, Brunvand M, et al (1993) Induction of alloantigen-specific hyporesponsiveness in human T lymphocytes by blocking interaction of CD28 with its natural ligand B7/BB1. J Exp Med 177: 165–173. 7678111 10.1084/jem.177.1.165 PMC2190874

[21] Thomas RM, Chunder N, Chen C, Umetsu SE, Winandy S, et al (2007) Ikaros enforces the costimulatory requirement for IL2 gene expression and is required for anergy induction in CD4+ T lymphocytes. J Immunol 179: 7305–7315. 18025173 10.4049/jimmunol.179.11.7305

[22] Rowell EA, Wang L, Hancock WW, Wells AD (2006) The cyclin-dependent kinase inhibitor p27kip1 is required for transplantation tolerance induced by costimulatory blockade. J Immunol 177: 5169–5176. 17015702 10.4049/jimmunol.177.8.5169

[23] Rowell EA, Walsh MC, Wells AD (2005) Opposing roles for the cyclin-dependent kinase inhibitor p27kip1 in the control of CD4+ T cell proliferation and effector function. J Immunol 174: 3359–3368. 15749868 10.4049/jimmunol.174.6.3359

[24] Boussiotis VA, Freeman GJ, Taylor PA, Berezovskaya A, Grass I, et al (2000) p27kip1 functions as an anergy factor inhibiting interleukin 2 transcription and clonal expansion of alloreactive human and mouse helper T lymphocytes. Nat Med 6: 290–297. 10700231 10.1038/73144

[25] Levin JZ, Yassour M, Adiconis X, Nusbaum C, Thompson DA, et al (2010) Comprehensive comparative analysis of strand-specific RNA sequencing methods. Nat Methods 7: 709–715. 10.1038/nmeth.1491 20711195 PMC3005310

[26] Kartashov AV, Barski A (2014) Wardrobe—an integrated system for analysis of epigenomics and transcriptomics data. bioRxiv: 012799 Available: http://biorxiv.org/content/early/2014/12/22/012799.abstract. 10.1186/s13059-015-0720-3 PMC4531538 26248465

[27] Dobin A, Davis CA, Schlesinger F, Drenkow J, Zaleski C, et al (2013) STAR: Ultrafast universal RNA-seq aligner. Bioinformatics 29: 15–21. 10.1093/bioinformatics/bts635 23104886 PMC3530905

[28] Anders S, Huber W (2010) Differential expression analysis for sequence count data. Genome Biol 11: R106 10.1186/gb-2010-11-10-r106 20979621 PMC3218662

[29] Chen J, Bardes EE, Aronow BJ, Jegga AG (2009) ToppGene Suite for gene list enrichment analysis and candidate gene prioritization. Nucleic Acids Res 37: W305–W311. 10.1093/nar/gkp427 19465376 PMC2703978

[30] Brownlie RJ, Zamoyska R (2013) T cell receptor signalling networks: branched, diversified and bounded. Nat Rev Immunol 13: 257–269. 10.1038/nri3403 23524462

[31] Rengarajan J, Mittelstadt PR, Mages HW, Gerth AJ, Kroczek RA, et al (2000) Sequential involvement of NFAT and Egr transcription factors in FasL regulation. Immunity 12: 293–300. 10755616 10.1016/s1074-7613(00)80182-x

[32] Schwartz RH (2003) T cell anergy. Annu Rev Immunol 21: 305–334. 12471050 10.1146/annurev.immunol.21.120601.141110

[33] Heissmeyer V, Macián F, Im S-H, Varma R, Feske S, et al (2004) Calcineurin imposes T cell unresponsiveness through targeted proteolysis of signaling proteins. Nat Immunol 5: 255–265. 14973438 10.1038/ni1047

[34] Pagano M, Tam SW, Theodoras AM, Beer-Romero P, Del Sal G, et al (1995) Role of the ubiquitin-proteasome pathway in regulating abundance of the cyclin-dependent kinase inhibitor p27. Science 269: 682–685. 7624798 10.1126/science.7624798

[35] Li L, Iwamoto Y, Berezovskaya A, Boussiotis VA (2006) A pathway regulated by cell cycle inhibitor p27Kip1 and checkpoint inhibitor Smad3 is involved in the induction of T cell tolerance. Nat Immunol 7: 1157–1165. 17013388 10.1038/ni1398

[36] Zheng Y, Zha Y, Driessens G, Locke F, Gajewski TF (2012) Transcriptional regulator early growth response gene 2 (Egr2) is required for T cell anergy in vitro and in vivo. J Exp Med 209: 2157–2163. 10.1084/jem.20120342 23129747 PMC3501351

[37] Zha Y, Marks R, Ho AW, Peterson AC, Janardhan S, et al (2006) T cell anergy is reversed by active Ras and is regulated by diacylglycerol kinase-alpha. Nat Immunol 7: 1166–1173. 17028589 10.1038/ni1394

[38] Safford M, Collins S, Lutz M, Allen A, Huang C-T, et al (2005) Egr-2 and Egr-3 are negative regulators of T cell activation. Nat Immunol 6: 472–480. 15834410 10.1038/ni1193

[39] Zheng Y, Zha Y, Gajewski TF (2008) Molecular regulation of T-cell anergy. EMBO Rep 9: 50–55. 10.1038/sj.embor.7401138 18174897 PMC2246614

[40] Collins S, Lutz M, Zarek PE, Anders R, Kersh GJ, et al (2008) Opposing regulation of T cell function by Egr-1/NAB2 and Egr-2/Egr-3. Eur J Immunol 38: 528–536. 10.1002/eji.200737157 18203138 PMC3598016

[41] Polyak K, Lee MH, Erdjument-Bromage H, Koff A, Roberts JM, et al (1994) Cloning of p27Kip1, a cyclin-dependent kinase inhibitor and a potential mediator of extracellular antimitogenic signals. Cell 78: 59–66. 8033212 10.1016/0092-8674(94)90572-x

[42] Powell JD, Bruniquel D, Schwartz RH (2001) TCR engagement in the absence of cell cycle progression leads to T cell anergy independent of p27Kip1. Eur J Immunol 31: 3737–3746. 11745394 10.1002/1521-4141(200112)31:12<3737::aid-immu3737>3.0.co;2-g

[43] Riella LV, Liu T, Yang J, Chock S, Shimizu T, et al (2012) Deleterious effect of CTLA4-Ig on a Treg-dependent transplant model. Am J Transplant 12: 846–855. 10.1111/j.1600-6143.2011.03929.x 22300534

[44] Xue H-H, Kovanen PE, Pise-Masison C, Berg M, Radovich MF, et al (2002) IL-2 negatively regulates IL-7 receptor alpha chain expression in activated T lymphocytes. Proc Natl Acad Sci U S A 99: 13759–13764. 12354940 10.1073/pnas.212214999 PMC129770

[45] Mazzucchelli R, Durum SK (2007) Interleukin-7 receptor expression: intelligent design. Nat Rev Immunol 7: 144–154. 17259970 10.1038/nri2023

[46] Liao W, Lin J-X, Leonard WJ (2013) Interleukin-2 at the crossroads of effector responses, tolerance, and immunotherapy. Immunity 38: 13–25. 10.1016/j.immuni.2013.01.004 23352221 PMC3610532

